# Linguistic Illusions Guide Eye Movement: Evidence From Doubling

**DOI:** 10.1007/s10936-023-10023-y

**Published:** 2023-10-12

**Authors:** Qatherine Andan, Peter Bex, Iris Berent

**Affiliations:** Department of Psychology, Northeastern University, 125 Nightingale Hall, 360 Huntington Ave., Boston, MA 02115 USA

**Keywords:** Amodal phonology, Eye movement, Phonology, Morphology, Doubling, Optimality theory

## Abstract

Across languages, certain phonological patterns are preferred to others (e.g., *blog* > *lbog*). But whether such preferences arise from abstract linguistic constraints or sensorimotor pressures is controversial. We address this debate by examining the constraints on doubling (e.g., *slaflaf*, generally, XX). Doubling demonstrably elicits conflicting responses (aversion or preference), depending on the linguistic level of analysis (phonology vs. morphology). Since the stimulus remains unchanged, the shifting responses imply abstract constraints. Here, we ask whether these constraints apply online, in eye movements. Experiment 1 shows that, in bare phonological forms, doubling is dispreferred, and correspondingly it elicits shorter fixations. Remarkably, when doubling signals morphological plurality, the aversion shifts into preference, in Experiment 2. Our results demonstrate for the first time that the constraints on doubling apply online. These findings are consistent with the hypothesis that phonological knowledge arises, in part, from an abstract linguistic source.

## Introduction

Every spoken language constrains the co-occurrence of sounds, such that some phonological sequences are preferred to others (e.g., *blog* > *lbog*). While the existence of phonological preferences is generally recognized, their source is unclear.

One explanation for linguistic preferences appeals to sensorimotor pressures on the channel of communication. For example, the dispreference for *lbog* could arise because such sequences exact higher demands on speech production or perception (e.g. MacNeilage, 2008; Pulvermüller & Fadiga, 2010). This is the *embodiment account* of language preferences.

Alternatively, linguistic preferences might arise from a grammatical source (e.g., McCarthy & Prince, 1993; Prince & Smolensky, 1993/2004; Berent, 2013). In this view, words are assigned structured representations in the minds of speakers, and these representations are constrained by abstract grammatical principles. Words that better abide by these principles are better-formed, and for this reason, they are preferred. Linguistic preferences, then, are governed by abstract linguistic principles, rather than sensorimotor demands. We refer to this account as the *abstraction* hypothesis.

The embodiment and abstraction accounts give rise to competing testable predictions. In the embodiment account, all things being equal, a single linguistic stimulus should always elicit the same linguistic response (either preference or aversion), as its sensorimotor demands are invariant. Thus, the mapping between the stimulus and the linguistic response ought to be one-to-one. The abstraction account, on the other hand, predicts that linguistic preferences arise from the abstract representations assigned to the stimulus, and, in principle, a single stimulus could be assigned multiple abstract representations. If so, it is conceivable that, much like ambiguous figures in vision, a single word could give rise to competing linguistic responses (preferences vs. aversion); the mapping between stimulus and linguistic response need not always be one-to-one.

One way to adjudicate between these contrasting explanations for language preferences is to determine whether a single stimulus with invariant sensorimotor properties can indeed give rise to competing responses. The critical question, then, is whether linguistic preferences can dissociate from sensorimotor pressures. This is our concern here.

### The Restrictions on Doubling

To adjudicate between the abstraction and embodiment accounts, past research explored the restrictions on linguistic doubling (Berent et al., 2016). Doubling is the repetition of a phonological constituent (XX, where X is a phonological constituent, e.g., the syllable ‘na’ in *banana*). Linguistic theory suggests that doubling is structurally ambiguous, as doubling is compatible with at least two distinct structural parses. This ambiguity matters because each such parse violates distinct linguistic constraints, and consequently, the two parses elicit conflicting linguistic responses (McCarthy, 1986; McCarthy & Prince, 1995; Inkelas, 2008; Berent et al., 2016; Urbanczyk, 2017).

At the phonological level, doubling is parsed simply as two repeated phonological elements (XX); here, the doubling has no bearing on meaning. For example, the repeated *na* in *banana* adds no meaning to *bana*; doubling here is purely phonological. Doubling, however, can also arise at the morphological level. For example, in *Manam*, the word *pana* ‘to run’ can be doubled, forming *panana* ‘to chase’ (Lichtenberk, 1983). Here, the change in form (the repetition of ‘na’) signals a change in the word’s meaning, and this morphological parse is called *reduplication*. Formally, reduplication is the copying (fully or partially) of a base, henceforth notated X{X}_copy_ (Berent et al., 2016).

The choice of the parse matters because each such parse violates distinct grammatical constraints. Phonological identity (XX) is banned by the Obligatory Contour Principle (OCP), a grammatical ban on identical elements (Leben, 1973; McCarthy, 1986). And indeed, identity is systematically avoided across languages (Suzuki, 1998). In contrast, when doubling is parsed as reduplication, the copy (X_copy_) is akin to a shadow—it is devoid of phonological substance (just as the ethereal shadow doesn’t count as another person). Accordingly, only one phonological element is present (rather than two), so the OCP is not violated. Furthermore, since the base ({X}) is shorter than the no-doubling control (XY), reduplication better satisfies DEP (dependent)—a constraint that bans the addition of phonological material (Prince & Smolensky, 1993)—and so {X}Xcopy is actually better formed than XY. In line with this analysis, reduplication is frequent across languages (Rubino, 2013).

Summarizing, then, doubling can be assigned two competing parses. At the phonological level, it is parsed as identity (XX), which is systematically dispreferred (due to the violation of the OCP). But at the morphological level, doubling is parsed as reduplication (as {X}X_copy_), and is actually preferred (due to the satisfaction of DEP). These competing parses and their consequences are summarized in Table 1.

**Table 1:** Linguistic principles constraining the two forms of doubling (adapted from Berent et al., 2016)

Grammatical parse	Representation	OCP	DEP
Phonological identity	*XX	✕	
	XY	✓	
Morphological reduplication	{X}X_copy_		✓
	XY		✕

### Experimental Investigations of Doubling

A series of experiments has shown that the conflicting preferences towards doubling are also evident in the behavior of individual speakers (Berent et al., 2016). In these experiments, participants were asked to make a forced choice between novel words that had doubling and no-doubling controls (e.g., *slaflaf* vs. *slafmat*). Results showed that participants’ preference shifted, depending on the implied level of linguistic analysis. When presented with bare phonological forms, English speakers systematically dispreferred doubling to controls (e.g. *slaflaf* < *slafmat*). But when doubling signaled plurality (e.g., *slaf* = one ball; *slaflaf* = a set of balls), doubling was instead systematically *preferred* (e.g. *slaflaf* > *slafmat*).

Berent and colleagues argued that, since the input that elicited these responses is unchanged, the shift in response—from aversion (in the phonological condition) to preference (in the morphological condition)—cannot be attributed to sensorimotor demands. Instead, the conflicting responses emerged because participants assign doubling competing grammatical parses (identity vs. reduplication, respectively).

Several observations support this possibility. First, doubling preferences only obtained when the meaning associated with doubling was semantically licit—i.e., when doubling signaled a homogeneous set of objects (e.g., several balls), but not for a heterogeneous set (e.g., a dog, a frog and a rattle). This finding is important because it suggests that English speakers prefer doubling only when a morphological parse is linguistically viable.

Second, participants projected the same doubling preferences even when the stimulus modality was changed. Here, participants—English speakers with no command of a sign language—were invited to choose between two novel signs in American Sign Language. Results showed, that when presented with bare phonological forms, English speakers dispreferred doubling (XX < XY), as they did for novel words. But when doubling in signs signaled morphological plurality, here, the doubling aversion turned into a preference (XX > XY), in line with the results for spoken language. The converging responses to spoken and signed language is consistent with an abstract grammatical source.

Third, these preferences (for both speech and sign) were constrained by the grammar of a speaker’s native language. For example, Hebrew uses reduplication productively, as part of its morphology. For example, the Hebrew morphology uses partial reduplication to mark diminution (e.g., *kelev* ‘dog’ → *klavlav* ‘puppy’), whereas English does not. These differences between English and Hebrew seem to affect how their speakers respond to signs. Indeed, when reduplication in sign signaled diminution (e.g., X = ball; XX = a little ball), Hebrew (but not English) speakers showed doubling preference (i.e., XX > XY). In contrast, when doubling signaled plurality, the doubling preference was found in English only (Berent et al., 2016). These results suggest that doubling preferences depend crucially on the morphological characteristics of a speaker’s native language. Subsequent work has expanded these results to other languages (Mandarin vs. Malayalam; Berent et al., 2020a, 2020b) and additional doubling structures (Berent, Bat-El, Brentari, Andan, & Vaknin-Nusbaum, 2020).

Taken together, these results suggest that (a) responses to a single stimulus (whose sensorimotor demands are invariant) can vary, depending on its putative grammatical parse; whereas (b) when two stimuli in distinct modalities share the same grammatical parse (e.g., identity), they elicit the same response, even though their sensorimotor demands are vastly different. This double dissociation between the sensorimotor demands and the linguistic responses suggests that linguistic preferences arise from principles that are abstract.

These conclusions, however, are limited by the fact that the findings obtain *offline* (i.e. in the form of acceptability ratings), and this casts doubt on both the nature of these principles and their scope. One concern is that the constraints on doubling are partly meta-linguistic. For example, the preference to mark semantic plurals by doubling may arise because of an iconic pressure to mark sets of multiple identical elements (e.g., many balls) by linguistic forms with identical elements (e.g., *slaflaf*). The failure of Hebrew and Mandarin speakers to apply this strategy already speaks against this possibility (Berent et al., 2016, 2020a, 2020b), and suggests that the constraints on doubling are indeed abstract and linguistic. Still, these conclusions are limited, inasmuch as it is unclear whether these putative linguistic principles play a role in natural language processing. A critical open question, then, is whether these constraints on doubling apply on-line.

### The Present Experiment

The present research thus examines whether the constraints on doubling affect eye movement on-line, in the natural course of language processing. As in previous research (Berent et al., 2016), in each trial, participants were asked to choose between two written words: one had doubling (e.g., *slaflaf*), and another was a no-doubling control (e.g., *slafmat*). In the phonological condition (Experiment 1), these words were presented as bare phonological forms; in the morphological condition (Experiment 2), the same words were presented as names for object sets, such that doubling potentially marked semantic plurality. As participants viewed these options, their eye movements were recorded. This allowed us to compare offline choice for doubling with on-line looking behavior.

A large literature demonstrates that skilled readers automatically decode phonological structure in silent reading (e.g. Leinenger, 2014; Lukatela & Turvey, 1994; Van Orden, 1987). Moreover, phonological and morphological processing is known to rapidly affect looking time, beginning within the first fifty milliseconds of a fixation to a written word (e.g., Slattery et al., 2006). Our question is whether restrictions on doubling likewise emerge rapidly, as expected of grammatical processes.

The *abstraction* hypothesis predicts that they do. If so, doubling preferences should apply online, in early language processing, possibly even within the first fixation. Furthermore, the pattern of online doubling preferences should shift, depending on the linguistic level of analysis implied by the context—phonology or morphology. At the phonological level (in Experiment 1) we expect to see evidence for doubling aversion, whereas at the morphological level (in Experiment 2), the aversion should change into a preference. Critically, these dynamic doubling preferences should obtain both in participants’ offline choices and in the pattern of their eye movement.

The predictions of the *embodiment* hypothesis are less clear. A strong embodiment view denies that abstract doubling preferences exist; if so, the doubling response should be only determined by the demands exacted by processing the stimulus, and since these demands are invariant, so should be the doubling response. Doubling response, then, should exhibit either aversion or preference, but not both. A weaker version might acknowledge that abstract preferences exist but deny that they can constrain on-line language processing; in this view, doubling preferences arise entirely from metalinguistic strategies that are presumably non-automatic, hence, late emerging. This second view predicts that doubling preference could potentially constrain offline behavior (i.e., in doubling choice) but not on-line looking time; and certainly not the first fixation. Here, we consider these two versions of this hypothesis; other possibilities are considered in the General Discussion.

Because the predictions of the embodiment hypothesis can vary vastly, this hypothesis is difficult to fully falsify. Our research strategy, then, is to first ask whether doubling preferences apply on-line, and rapidly—in a manner that is consistent with *abstraction*. Once we have demonstrated that is the case, in the General Discussion, we turn to discuss how the *abstraction* and *embodiment* hypotheses each fare against these results.

## Experiment 1

In Experiment 1, each trial featured a picture of a single novel object, along with a pair of words (e.g., *slaflaf* vs. *slafmat*). Since doubling here is utterly unrelated to meaning, we expect participants to parse doubling as phonological identity (as XX). Consequently, participants should be *less* likely to choose doubling compared to controls (e.g., *slaflaf* < *slafmat*). We also expect this doubling aversion to emerge in the pattern of eye movement.

How doubling aversion would manifest in terms of looking behavior is uncertain a priori. One possibility is that the phonological ill-formedness of doubling could be associated with high processing costs, resulting in longer looking times (including first fixation durations and total looking time) and more frequent regressive looks for words with doubling compared to controls. Alternatively, the doubling aversion could lead to a parallel gaze aversion, resulting in shorter looking times and less overall time spent looking at words with doubling relative to controls. For our purposes, however, the main question is *whether* sensitivity to doubling arises online. The *direction* of these effects is of lesser importance.

Because our stimuli are written, and because English reading proceeds from left to right, we further expect that looking behavior might be affected by a word’s relative position on the screen (i.e. left vs. right). The direction of the position effect is likewise unknown. One possibility is that left words benefit from their primacy, resulting in greater sensitivity to their linguistic structure (relative to right words). Alternatively, it is possible that it is instead right words that are privileged, as their processing can be primed by the prior processing of their left counterparts. To account for these positional effects, our analyses probe the effects of both word type (doubling vs. no doubling) and position (left vs. right).

### Methods

#### Participants

Thirty-three native English speakers, students at Northeastern University, participated in Experiment 1. Data from three additional participants were excluded because of data loss (loss of over 50% of the eye tracking data; in two participants, this was due to eye tracker calibration failure, and, in one case, programming error). Sample size in Experiments 1–2 was set to match that in Berent et al.’s (2016) studies.

All participants signed informed consent forms according to local IRB guidelines; they were debriefed following the conclusion of the experiment and received course credit as compensation for their participation.

#### Materials

The experimental materials were printed words of two types: doubling (e.g. *slaflaf*) and no-doubling controls (e.g. *slafmat*). All items had a CCVC-CVC structure (C = consonant; V = vowel), and they were arranged in pairs. Pair members were matched for length and the initial syllable, i.e., the base (e.g. *slaf*), and differed on the second syllable. In doubling words, the second syllable copied the second, third and fourth letters of the base, (C_1_C_2_VC_3_–**C**_**2**_**VC**_**3**_, e.g. *slaflaf*), whereas for the no-doubling controls, the second syllable was different from the first (C_1_C_2_VC_3_–**C**_**4**_**VC**_**5**_, e.g. *slafmat*). The experiment featured a total of 30 such unique pairs (for the full list of stimuli, see Appendix 1).

These words pairs were presented to participants in two blocks. Each block featured all 30 pairs, counterbalanced for Type (doubling vs. no doubling) and side (left vs. right); the two blocks differed on the position of the pair members (left vs. right). Trial order was randomized within block; the same trial order was presented to all participants.

Each trial presented a word pair along with a picture of a single object, whose name is likely unfamiliar to participants (e.g. obscure kitchen implements). Each word pair was consistently associated with the same unique object throughout the two blocks.

#### Procedure

The experiment was presented to participants on a computer monitor. Participants initiated the trial by pressing the space bar. They next saw a novel object (256 × 256 pixels), presented at the center of a white screen. Above the object was a text prompt (“What is the best name for this object?”); below the object, there was a text rectangle (“Click to Proceed”; all text was presented in a *Letter Gothic* 32pt font). Once participants responded to this display (by clicking anywhere on the screen), the rectangle was replaced with a pair of novel words, centered horizontally below the object. As soon as the words appeared, the eye tracker began recording participants’ eye movements. Participants were given up to five seconds to choose among the two words (by clicking on one of the two words), and once they did, the eye tracking was terminated.

This upper bound on response was set in order to encourage participants to respond rapidly. To this end, in Experiments 1–2, we did not allow participants to respond past the five-second limit. Slow responses (above five seconds) triggered a warning message (“Trial Timed Out–Try not to overthink your answer!”); these slow responses were not collected, but the corresponding eye movement data were analyzed.

**Fig. 1:**
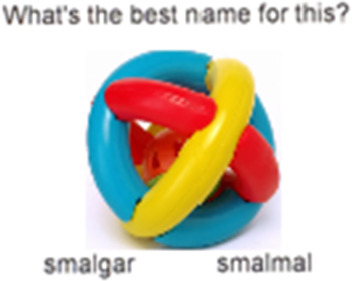
An example of an experimental trial. *Ball photo credit*: FreeDigitalPhotos.net, image creators: Suat Eman

The eye tracker was a Tobii EyeX, which recorded eye movement data at a rate of 60 Hz. The experiment was run using Psychtoolbox (Brainard, 1997) in Matlab, at a screen resolution of 1920 × 1080 pixels, using the Tobii SDK developed for Matlab (Gibaldi et al., 2017) (Fig. 1).

#### Eye Movement Data Collection

Prior to the start of the experiment, each participant was calibrated on a Tobii EyeX eye tracker using the Tobii software package’s native calibration sequence. Participants were seated at a viewing distance of 60 cm from the eye tracker. Nine fixation points spaced evenly throughout the screen were used to judge the calibration’s success—calibration was deemed successful if all nine of these points were calibrated successfully, within about one degree of visual angle’s worth of error. Participants that could not calibrate all nine points successfully were recalibrated a maximum of two more times—if the third calibration was sub-optimal, the experimenter made note of the inconsistent calibration and proceeded with the experiment as usual. Because of this, the data for some participants (two in Experiment 1) were excluded from any further analysis due to inconsistent or failed calibration, resulting in a substantial loss of eye tracking data (> 50%). See Appendix 2 for data pre-processing and processing methods.

#### Measure of Doubling Preference

Doubling preferences were measured by both offline choice (i.e., the proportion of doubling responses; see Sect. "Offline Choice") and on-line looking behavior (Sect. "On-Line Measures").

On-line behavior was gauged by three measures. *First fixation durations* were the duration of the first fixation to each word, where a fixation was any continuous look longer than 100 ms.^1^ *Total looking time* was the total time spent looking at a word throughout a trial (including both fixations and shorter looks). *Regressive fixations* were the number of word fixations occurring after the first fixation.

### Results

#### Offline Choice

As predicted, when doubling had no meaning, participants showed doubling aversion. They selected doubling on 0.35 of the trials. A binomial test showed that this proportion of doubling choice was significantly lower than chance (M = 0.35, Z =  − 12.37, *p* < 0.001). These results replicate previous findings (Berent et al., 2016, 2020a, 2020b) showing that phonological doubling is dispreferred. Our main interest, however, is whether this aversion applies on-line, in looking behavior.

#### On-Line Measures

**Fig. 2:**
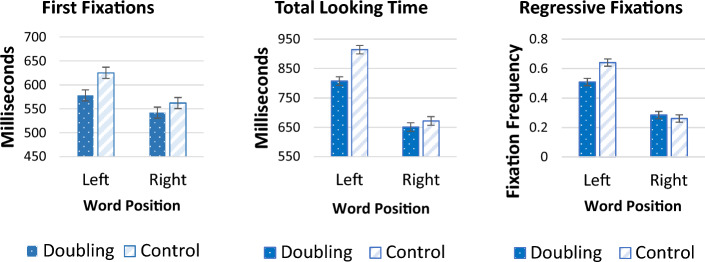
First fixation durations, total looking times, and regressive fixations as a function of word position and type in Experiment 1. For these and all subsequent figures, error bars are 95% confidence intervals for the interaction

Figure 2 plots the participants’ looking time behavior. An inspection of the means suggests that left words elicited longer looking time than right words. Critically, words with doubling elicited shorter looking time than controls, especially when these words were presented on the left side. Remarkably, these effects were evident already in the first fixation.

We next evaluated these observations via 2 Type (doubling vs. control) × 2 Side (left vs. right) repeated measures ANOVAs, conducted over each of three looking time measures (first fixation durations, total looking time and regressive fixations). Each such analysis was conducted using both participants and items as random effects. We note that, although the mean regressive fixations are smaller than 1, the relevant scale (the number of regressive fixations) is continuous (rather than binary), so the ANOVA is in order.

##### First Fixation Durations

The ANOVA of first fixation durations yielded a significant main effect of Side (F_1_ (1, 32) = 6.53, *p* = 0.016, η^2^_partial_ = 0.17; F_2_ (1, 29) = 24.706, *p* < 0.001, η^2^_partial_ = 0.46), as left words elicited longer first fixations than right words. Critically, there was also a main effect of Type (F_1_ (1, 32) = 10.16, *p* = 0.003, η^2^_partial_ = 0.24; F_2_ (1, 29) = 10.42, *p* = 0.003, η^2^_partial_ = 0.26), as doubling elicited shorter looks than controls. The interaction was not significant (F_1_ (1, 32) = 2.85, *p* = 0.10, η^2^_partial_ = 0.08; F_2_ < 1, η^2^_partial_ = 0.03).

Thus, participants showed aversion to doubling already within the first fixation. These results suggest that doubling affects online processing, and that this effect manifests itself in early language processing.

##### Total Looking Time

An inspection of the means suggests that, unlike the first fixation, for total looking time, doubling preference emerged for left words only.

The ANOVA yielded a significant effect of Side (F_1_ (1, 32) = 93.89, *p* < 0.001, η^2^_partial_ = 0.75; F_2_ (1, 29) = 329.596, *p* < 0.001, η^2^_partial_ = 0.92) and word Type (F_1_(1, 32) = 18.58, *p* < 0.001, η^2^_partial_ = 0.37; F_2_ (1, 29) = 19.242, *p* < 0.001, η^2^_partial_ = 0.40). The interaction was now significant (F_1_ (1, 32) = 17.57, *p* < 0.001, η^2^_partial_ = 0.35; F_2_ (1, 29) = 4.544, *p* = 0.042, η^2^_partial_ = 0.14).

We next tested for the effect of doubling for left- and right- words using planned contrasts. For left words, doubling elicited shorter total looking times compared to control words (t_1_ (32) = 7.37, *p* < 0.001, d = 0.47; t_2_ (29) = 3.65, *p* = 0.001, d = 1.01). This, however, was not the case for right words (t_1_ (32) = 1.44, *p* = 0.160, d = 0.11; t_2_ (29) = 0.63, *p* = 0.53, d = 0.25).

##### Regressive Fixations

The ANOVA revealed significant main effects of Side (F_1_ (1, 32) = 90.69, *p* < 0.001, η^2^_partial_ = 0.74); F_2_ (1, 29) = 324.93, *p* < 0.001, η^2^_partial_ = 0.92) and word Type (F_1_ (1, 32) = 7.02, *p* = 0.012, η^2^_partial_ = 0.18); F_2_ (1, 29) = 9.76, *p* = 0.004, η^2^_partial_ = 0.25). There was also a significant interaction between Side and word Type (F_1_ (1, 32) = 20.15, *p* < 0.001, η^2^_partial_ = 0.39); F_2_ (1, 29) = 26.66, *p* < 0.001, η^2^_partial_ = 0.48).

Planned contrasts revealed that on the left, words with doubling were re-read significantly less often than control words (t_1_ (32) = 5.39, *p* < 0.001, d = 0.82; t_2_(29) = 6.20, *p* < 0.001, d = 0.99). This however was not the case for words presented on the right (t_1_ (32) =  − 0.96, *p* = 0.34, d =  − 0.14; t_2_ (29) =  − 1.10, *p* = 0.28, d =  − 0.20).

### Discussion

The results for Experiment 1 show that when doubling reflects phonological identity (i.e., it signals no change in meaning), English speakers are systematically averse to doubling. Moreover, this doubling aversion is evident both offline and in on-line looking behavior.

The offline measure showed that English speakers are less likely to choose words with doubling relative to controls (e.g., *slaflaf* < *slafmat*); this result replicates the previous findings of Berent et al. (2016). Critically, our present results are the first to show that this aversion applies on-line, and it emerges already within the first fixation.

Words with doubling elicited shorter first fixations, shorter total looking time, and fewer regressive fixations relative to no-doubling controls. For first fixations, these effects emerged irrespective of whether doubling was presented on the left on or the right; the later processing measures (total looking time and regressive fixations) showed this doubling aversion only when doubling was presented on the left. This effect of side possibly arose because reading English proceeds from left to right. Consequently, participants may have attended to left words more than to right ones, and correspondingly, might have been more attentive to their internal structure.

The fact that these three different measures of looking time converge, and that they further mirror the offline choice, is consistent with the possibility that participants’ doubling aversion leads to a parallel aversion of the gaze (i.e., dis-preference begets shorter looks). This finding might seem at odds with previous results on morphological and syntactic processing, where ungrammaticality often elicits longer looking time (i.e., processing cost; see Ni et al., 1998; Braze et al., 2002). However, the relation between looking time and grammaticality is far from straightforward. For example, in the infant literature, grammaticality has been shown to elicit conflicting outcomes (e.g., see Gomez & Gerken, 1999; Marcus et al., 1999; Soderstrom, Seidl, Nelson & Jusczyk, 2003). Moreover, to our knowledge, no previous research has used eye movement to gauge the role of grammatical *phonological* structure, specifically. Thus, a priori, we see no basis to predict how looking times should pattern in the present experiment.

If this interpretation is correct, and the results from Experiment 1 indeed show doubling aversion, then the question arises as to why such aversion obtains here. On the *abstraction* account, doubling aversion results from the linguistic ban on phonological identity (see Table 1). But as noted, these findings could also arise for other reasons. For example, doubling could be dispreferred on-line because words with doubling are more difficult for the visual system to encode (Kanwisher, 1987), in line with the *embodiment* explanation. Another possibility is that doubling aversion arises because the statistical properties of doubling are less frequent in English.

To adjudicate between these possibilities, Experiment 2 examines responses to the same words in a morphological context—when doubling signals plurality. If on-line doubling aversion is only driven by nonlinguistic sources—either the demands on the visual system, or the statistical properties of words—then the same doubling aversion should reappear. But if these results are constrained by the linguistic parse, then once doubling is parsed morphologically, the doubling preferences—both offline and on-line—should also shift.

## Experiment 2: Morphological Condition

In Experiment 2, doubling was presented to signal plurality—a systematic change in meaning from singular to plural. To this end, each trial consisted of two steps. In the first step, participants saw the doubling base (e.g., *slaf*, the base of *slaflaf*) paired with a single object; participants confirmed that they had registered the name by typing it back into the computer. In the second step, they saw a set of 3–5 objects, together with two word options (e.g., *slaflaf* vs. *slafmat*). Participants were asked to choose which word made a better name for the object set, and meanwhile, their looking behavior was recorded.

In this experiment, then, doubling (in form) signals a change in meaning (plurality), so doubling is potentially a morphological operation. And when doubling is parsed morphologically, as reduplication (formally: {X}X_copy_), doubling should be actively preferred to controls (e.g., *slaflaf* > *slafmat*). If doubling preferences are constrained by linguistic principles, then in Experiment 2, doubling preference should shift from aversion (in Experiment 1) to preference.

To determine whether the shift is indeed due to morphology, Experiment 2 further varied the semantic properties of reduplication. We reasoned that a morphological parse requires licit links between form (e.g., *slaf → slaflaf*) and meaning: here, semantic plurality (e.g., one object → an object set). Semantic plurality, however, only applies to homogeneous sets of the same kind. For example, if *slaf* is a ball, then, to mark plurality, *slaflaf* ought to be linked with a homogeneous set of balls, but not with a heterogeneous set of objects of different kinds (e.g., a ball, a pacifier, a rattle). To determine whether the assignment of a morphological parse is constrained by semantic legality, our experiment thus examined the doubling preferences in two conditions. In the licit condition, all objects were of the same type as the initial base object (e.g., a set of ball-like objects). The illicit condition, by contrast, featured a heterogenous set of objects (e.g., a ball, a pacifier, a rattle). If doubling preferences are constrained by linguistic principles, then speakers should assign a morphological reduplicative parse only in the licit—but not in the illicit—condition. Accordingly, the preference for the reduplicative form (e.g., *slaflaf* > *slafmat*) should obtain only for licit plurals, not for illicit ones.

Altogether, then, in Experiment 2, we expect a dramatic shift in doubling preference from aversion (in Experiment 1) to a preference in the licit (but not illicit) condition. As in Experiment 1, we expect doubling preference to emerge both in offline choice and on-line behavior. Finally, looking behavior (and the doubling preference reflected by this behavior) could further be modulated by a word’s position on the display (left vs. right).

### Methods

#### Participants

A new group of 34 native English speakers participated in Experiment 2. Six additional participants were tested, but removed from the analysis as a result of significant data loss—three due to inconsistent calibration (loss of > 50% of the data), and three as a result of programmatic error. Participants took part in the experiment in partial fulfillment of course credit. All participants signed informed consent forms and were debriefed as per local IRB guidelines.

#### Materials and Procedure

Experiment 2 featured the same word pairs from Experiment 1. Trial structure, however, now involved two steps.

Participants first saw a picture of a single object along with its potential name (e.g., This is a *slaf*!) and a question (“What is the name of this object?”) presented under the object. The object and its name appeared on the left side of the screen. Participants were directed to type the name of the object, and they could view their response.

Once participants responded correctly, a set of objects appeared (on the right side of the screen), along with the prompt “What is the best name for these?” A rectangle below the set of objects said, “Click to Show Names”. Once the participant clicked on the rectangle, it was replaced with a pair of words (in the same location), and the eye-tracker began recording the participant’s eye movements. One word had doubling (e.g. *slaflaf*) and the other was a no-doubling control (e.g. *slafmat*).

Participants’ task was to choose which word made a better name for the set of objects by clicking on their choice. Participants had a maximum of five seconds to respond. As in Experiment 1, slow responses (over five seconds) triggered a warning message (“Trial Timed Out – Try not to overthink your answer!”, presented for two seconds). These slow responses were included in the analysis of looking time, but the choice data were not collected.

**Fig. 3:**
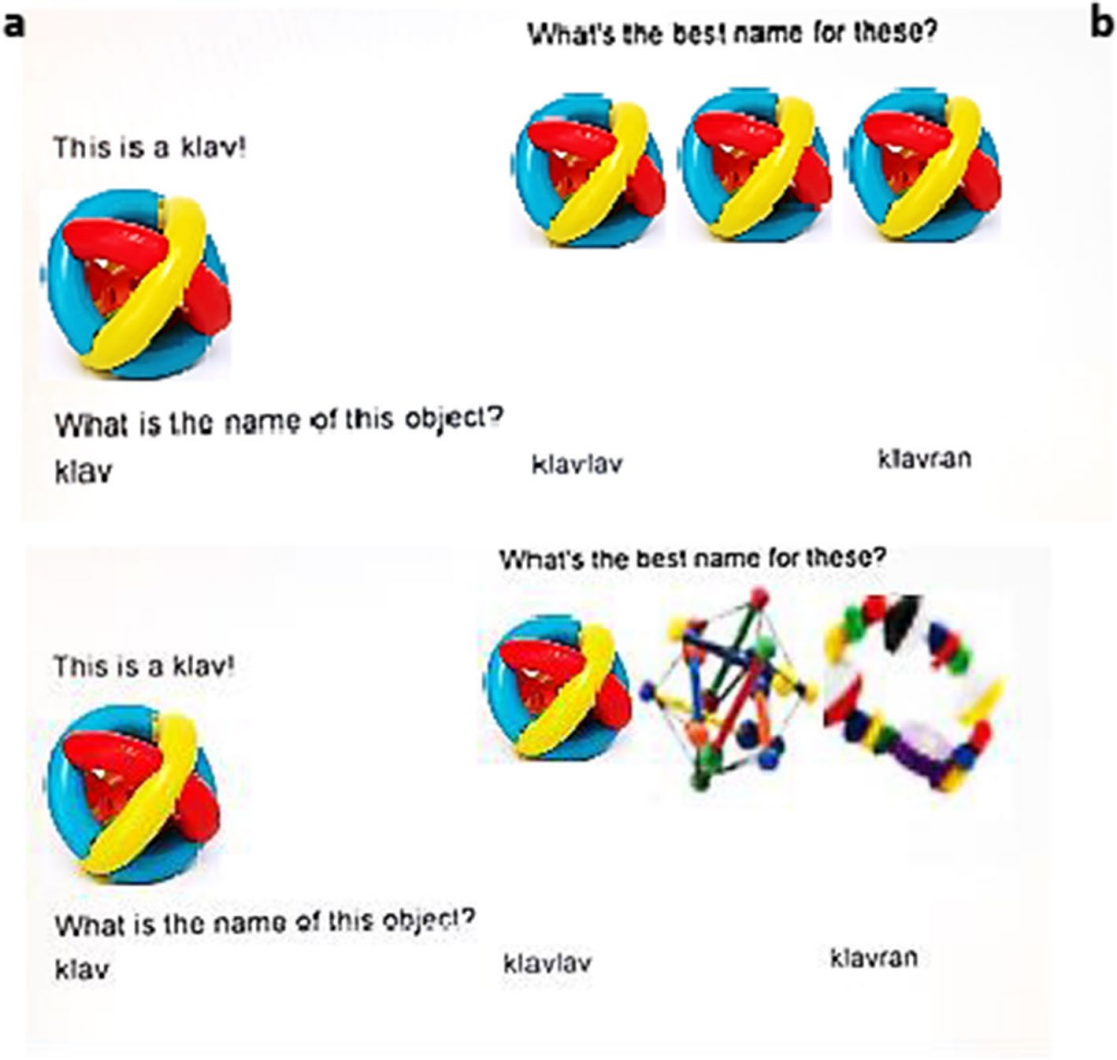
An example of a licit trial **a** and illicit trial **b**. *Ball photo credit*: FreeDigitalPhotos.net, image creator: Suat Eman. Images of the other objects are licensed under Creative Commons (modified)

In addition to word type (doubling or not), Experiment 2 further manipulated the semantic legality of the object set associated with the word pair. In the licit condition, all objects in the set were of the same kind as the base object (see Fig. 3a); in the illicit condition, the objects were of several different kinds. One of these kinds invariably included the base; the other kinds had comparable shape and color (Fig. 3b).

The 30 word pairs were arranged in two lists, assigned to the licit and illicit conditions. Because the number of pairs was uneven, it was not possible to fully cross the list for both word type (doubling vs. control) and position (left vs. right). As a result, the licit plural condition featured 16 trials with doubling on the left and 14 trials with doubling on the right; in the illicit plural condition, there were 14 trials with doubling on the left and 16 trials with doubling on the right. Licit and illicit plural trials were mixed randomly (within-block) and presented to participants in a single pre-randomized order using the PsychToolbox experiment software package (Brainard, 1997) in MATLAB.

### Results

The results for licit and illicit plural trials will be described separately, as they address different questions. We begin with the results for licit plural trials.

#### Licit Plural Trials

##### Offline Choice

As in Experiment 1, we first inspected the offline doubling choice. Doubling was chosen on 0.58 of the trials, and this choice was significantly above chance, as determined by a binomial t-test (Z = 4.63, *p* < 0.001). Thus, once doubling signaled plurality, participants preferred words with doubling over no-doubling controls. Our main interest, however, is in the three measures of online looking time, described below.

##### Online Measures

An inspection of the means suggests that, when doubling was presented on the right, it now elicited longer first fixations and longer looking time. Additionally, doubling further elicited more regressive fixations, and that was the case regardless of side.

**Fig. 4:**
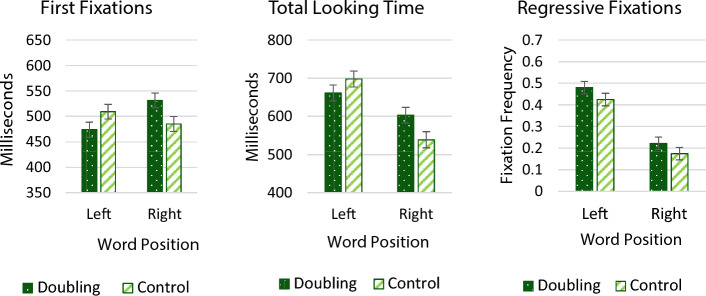
First fixation durations, total looking times, and mean number of regressive fixations as a function of word position and word type (licit plural trials)

We next evaluated the results for each of the three looking time measures using 2 (Side) X 2 (word Type) ANOVAs. The analysis by participants used a repeated measure design; across items, the analysis used a between items design (due to the uneven number of items in the list) (Fig. 4.)

##### First Fixation Durations

The ANOVAs yielded no significant main effect of Side (F_1_ < 1, η^2^_partial_ = 0.02; F_2_ (1, 29) = 1.36, *p* = 0.25, η^2^_partial_ = 0.02) or word Type (both Fs < 1 and η^2^_partial_ < 0.005). However, the Side × Type interaction was significant (F_1_ (1, 33) = 16.32, *p* < 0.001, η^2^_partial_ = 0.33; F_2_ (1, 29) = 9.962, *p* = 0.003, η^2^_partial_ = 0.15).

Planned contrast revealed that on the left, words with doubling elicited shorter first fixations than controls, an effect significant by participants (t_1_ (33) = 2.47, *p* = 0.019, d = 0.47) and marginally so by items (t_2_ (56) = 1.90, *p* = 0.063, d = 0.91). For right words, in contrast, doubling elicited significantly *longer* first fixations (t_1_ (33) =  − 3.25, *p* = 0.002, d =  − 0.39; t_2_ (56) = 2.58, *p* = 0.013, d =  − 0.79).

##### Total Looking Time

The ANOVA yielded a reliable main effect of Side (F_1_ (1, 33) = 31.10, *p* < 0.001, η^2^_partial_ = 0.49; F_2_ (1, 29) = 25.07, *p* < 0.001, η^2^_partial_ = 0.31), as left words elicited longer looking time than right words. The main effect of word Type was not significant (both Fs < 1). The interaction, however, was significant (F_1_ (1, 33) = 11.89, *p* = 0.002, η^2^_partial_ = 0.26; F_2_ (1, 29) = 5.18, *p* = 0.03, η^2^_partial_ = 0.08).

Planned contrasts showed that overall looks at left words did not differ reliably by their structure (t_1_ (33) = 1.78, *p* = 0.08, d = 0.26; t_2_ (56) = 1.13, *p* = 0.27, d = 0.43). For right words, however, words with doubling elicited significantly longer looking times than control words (t_1_ (33) =  − 3.10, *p* = 0.003, d =  − 0.42; t_2_ (56) =  − 2.10, *p* = 0.04, d =  − 0.74).

##### Regressive Fixations

The ANOVA yielded a reliable main effect of Side (F_1_ (1, 33) = 63.11, *p* < 0.001, η^2^_partial_ = 0.66; F_2_ (1, 29) = 81.99, *p* < 0.001, η^2^_partial_ = 0.59), as regressions were more likely for left- relative to right words. The main effect of word Type approached marginal (F_1_ (1, 33) = 3.18, *p* = 0.083, η^2^_partial_ = 0.09; F_2_ (1, 29) = 3.42, *p* = 0.07, η^2^_partial_ = 0.06), as doubling tended to elicit more regressive fixations than controls. The interaction was not significant (all F’s < 1).

Summarizing, when doubling lawfully signaled plurality, doubling was more likely to be chosen (offline), and, when presented on the right, it was associated with longer first fixations and longer looking time than controls. Across sides, doubling was further associated with more frequent regressive eye movements than controls, although this trend was not fully reliable. One notable exception to this pattern was the first fixations to left words, as here, doubling was less likely to attract first fixations; we will return to discuss this pattern in the Discussion.

#### Illicit Plural Trials

##### Offline Choice

When the link between the base and the reduplicative form was semantically illicit, there was no evidence for doubling preference. In fact, doubling choice (*P* = 0.409) was significantly below chance, as determined by a binomial t-test (Z =  − 5.75, *p* < 0.001). This doubling aversion to illicit plurals stands in stark contrast to the doubling preference found in the licit plural condition.

##### On-Line Measures

**Fig. 5:**
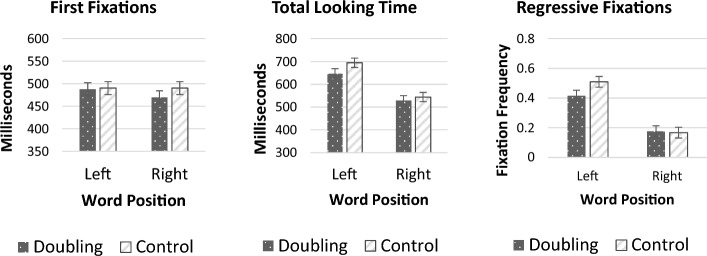
First fixations, total looking times, and regressive fixations as a function of word type and position (illicit plural trials)

An inspection of the online looking behavior (Fig. 5) likewise found no hint of a doubling preference. This does not seem to arise because the illicit condition elicited abnormal looking behavior. As in previous conditions, left words elicited the expected increase in looking time and first fixation. But there was no evidence for doubling preference. In fact, when doubling was presented on the left, doubling seemed to only be associated with fewer regressive fixations.

##### First Fixation Durations

An ANOVA found no significant main effect of Side or interaction (all Fs < 1). The effect of word Type was likewise not significant (F1 < 1; F_2_ (1, 29) = 1.33, *p* = 0.25, η^2^_partial_ = 0.02).

##### Total Looking Time

An ANOVA found a significant effect of side only (F_1_ (1, 33) = 68.51, *p* < 0.001, η^2^_partial_ = 0.67; F_1_ (1, 29) = 43.67, *p* < 0.001, η^2^_partial_ = 0.44), such that looking times were longer for left words. There was no effect of word type (F_1_ (1, 33) = 1.76, *p* = 0.19, η^2^_partial_ = 0.05; F_2_ (1, 29) = 3.01, *p* = 0.09, η^2^_partial_ = 0.05), and no interaction (F_1_ (1, 33) = 1.27, *p* = 0.26, η^2^_partial_ = 0.04; F_2_ < 1).

##### Regressive Fixations

The ANOVA only yielded a significant effect of Side (F_1_ (1, 33) = 71.96, *p* < 0.001, η^2^_partial_ = 0.69; F_2_ (1, 56) = 131.65, *p* < 0.001, η^2^_partial_ = 0.70). There was no significant effect of word Type (F_1_ = 2.31, *p* = 0.14, η^2^_partial_ = 0.07; F_2_ = 2.75, *p* = 0.10, η^2^_partial_ = 0.05). However, there was a marginal interaction between Side and word Type (F_1_ (1, 33) = 4.10, *p* = 0.051, η^2^_partial_ = 0.11; F_2_ (1, 29) = 3.99, *p* = 0.05, η^2^_partial_ = 0.07).

Planned comparisons showed that, for left words, doubling was less likely to attract regressive fixations than controls (t_1_(33) = 2.62, *p* = 0.013, d = 0.37; t_2_(56) = 2.59, *p* = 0.012, d = 0.79). This, however, was not the case on the right (both ts < 1).

These on-line measures, similar to the offline results, show that when a morphological interpretation of doubling was blocked by violating semantic preconditions for plurality, no doubling preference emerged.

### Discussions

The results from Experiment 2 show that when doubling lawfully signals plurality, doubling is systematically preferred, and this preference is evidence in both offline choice and on-line looking behavior. Offline, participants now were more likely to choose words with doubling as names for objects sets.

On-line, we found that when doubling was presented on the right, it now elicited longer first fixation durations and longer total looking times. Across sides, there was also a trend towards more frequent regressive fixations at words with doubling relative to controls. As expected, this preference only obtained when the link between the base and doubling was semantically licit: there was no hint of a doubling preference in the illicit condition.

These results seem to suggest that, when doubling signaled morphological plurality, participants now opted for a reduplicative parse, and, since this parse is well-formed, doubling was preferred. This proposal, however, is faced with two challenges. First, why did doubling preference only emerge on the right? Second, why did left words elicit *shorter* first fixations?

**Fig. 6:**
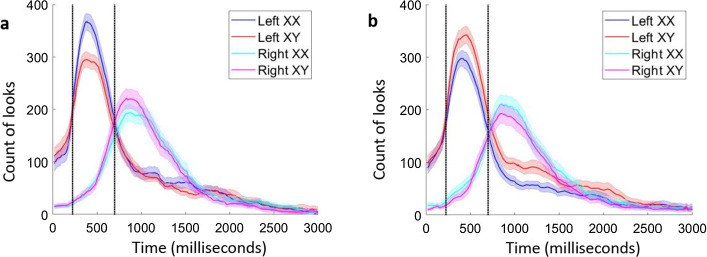
Counts of looks at doubling (XX) and control (XY) words over time for **a** licit and **b** illicit trials, respectively. Left words (blue and red lines) and right words (cyan and magenta) are plotted separately. Shaded regions show one standard error (SE of the count, $\sqrt{n}\times SD$) above and below the count (Color figure online)

We suggest that both findings arise from structural priming. Since reading English proceeds from left to right, with left words read first (as our data indeed show; see Fig. 6 below), words presented on the right can be primed by processing the left word. This priming is especially important in the morphological condition, as here, participants not only need to discover that doubling has internal structure, but further that this formal structure is systematically linked to the change in meaning. In particular, to discover that *slaflaf* is reduplicative (i.e., {X}X_copy_), readers must note that *slaflaf* includes the base *slaf.*

$$\begin{aligned} &(1)\quad {\text{Trial structure when doubling is on the left (a) vs. right (b).}}\\ & {a. \;Doubling \; on \; the \; left} \quad\quad  \quad {b. \; Doubling \; on \; the \; right} \\ & {\mathbf{slaf}} {\text{laf}} \qquad {\text{slafmat}} \qquad\qquad {\mathbf{slaf}}  {\text{mat}}  \qquad\qquad  {\mathbf{slaf}} {\text{laf}}\\ &.\end{aligned}$$

When doubling is presented on the right (1b), this information can be gleaned from the previous processing of the control, *slafmat* (on the left), and this encounter can make the base of *slaflaf* “pop out”. This structural priming, however, is not available when doubling is encountered first, on the left (1a). And if participants struggle to assign a reduplicative morphological parse, then they will see doubling as purely phonological identity, and doubling should be dispreferred (as in Experiment 1). Altogether, then, the priming account explains both why doubling preference (aided by a reduplicative parse) is more likely for right words, and concurrently, why on the first fixation left words elicit doubling aversion.

One puzzle, however, is still outstanding. If left words were less amenable for a reduplicative parse during the first fixation, how, then, did participants end up parsing them: did they parse them as identity (thereby triggering doubling aversion), or were they ultimately able to assign a reduplicative parse (hence, triggering a doubling preference)? Moreover, to the extent a reduplicative parse was ultimately assigned, how long did it take participants to arrive at this parse: was it only given after extensive deliberate processing, perhaps informed by metalinguistic strategies, or was it still assigned early on in the reading process (even if not on the first fixation)?

To address this question, we next inspected the dynamic pattern of looking time throughout the licit trials (see Fig. 6a); for comparison, we also present the looks at illicit trials. Unlike the first fixation durations, these graphs capture all looks, irrespective of whether they occur on the first fixation. An inspection of the looking trajectory shows that early on within the trial (within the first 250 ms), there were indeed fewer looks at the left XX words; this is consistent with the pattern of first fixation data on the left. Remarkably, however, Fig. 6a further shows that immediately thereafter, this pattern reverses—and for the window of time between 250 and 750 ms (between the black vertical lines), left words with doubling elicit vastly more looks than controls.

These results are consistent with the possibility that, as soon as participants re-fixated on the left words, they were immediately attracted to doubling, suggesting a doubling preference. Moreover, this behavior only emerged when the reduplicative parse was licit. An inspection of the illicit condition (Fig. 6b) within the same time window actually shows the reverse: an aversion of doubling.

Altogether, then, this analysis suggests that the assignment of a reduplicative parse was constrained by word position. Left words were not immediately assigned a reduplicative parse (on the first fixation), but they most likely were given one immediately thereafter—within the first 500 ms of reading. For right words, however, reduplication was preferred already within the first fixation, possibly because these words benefited from structural priming by their left counterparts. These results further contrast with the findings of Experiment 1, where doubling was consistently dispreferred. As such, our results show that doubling elicits conflicting responses, depending on the linguistic level of analysis—phonology vs. morphology.

## General Discussions

This research sought to unveil the source of linguistic preferences. We asked whether linguistic preferences are solely the product of sensorimotor pressures, or whether abstract linguistic principles could likewise play a role. We further explored whether such putative abstract linguistic constraints shape early language processing—in on-line looking behavior.

The results reported here suggest that doubling preferences of English speakers can demonstrably dissociate from the sensorimotor demands of the stimulus, inasmuch as a single stimulus can elicit conflicting responses—either aversion or preference. These dissociations are consistent with previous offline results (Berent et al., 2016; Berent, Bat-El, & Vaknin-Nusbaum, 2016; Berent, Bat-El, Brentari, Andan, & Vaknin-Nusbaum, 2020; Berent et al., 2020a, 2020b). Our present experiments, however, are the first to show that the constraints on doubling further shape online language processing, and they are evident already at the earliest stages of looking behavior.

In Experiment 1, we found that, when doubling has no link to meaning (i.e., doubling signals phonological identity), doubling is systematically dispreferred. This doubling aversion was observed not only offline, in participants’ tendency to avoid choosing doubling (relative to control words), but also on-line, in their eye movements. Doubling elicited shorter first fixations, and, when doubling was presented on the left, doubling also elicited shorter total looking times and fewer regressive fixations relative to controls.

We suggest that this doubling aversion arose because, at the phonological level, doubling is parsed as identity, and identity is banned by the grammar. To further test this possibility, Experiment 2 examined whether the doubling preference could shift when the context favors a different level of analysis. Here, doubling lawfully signaled plurality (e.g., *slaf* a ball; *slaflaf*, a set of balls)—consistent with a morphological reduplicative parse. And since reduplication better satisfies grammatical constraints (see Table 1), here, we expected a doubling preference.

As predicted, in the licit condition, doubling was indeed preferred. As in Experiment 1, this response was evident both offline (in the doubling choice) as well as in the on-line looking behavior. Now, when doubling was presented on the right, doubling elicited *longer* first fixations and longer looking time. Moreover, participants were more likely to re-read doubling words relative to controls (irrespective of their position, left or right).

As detailed in the Discussion of Experiment 2, right words might have been more susceptible to a morphological parse because they were primed by the previous processing of the left word. For this reason, their reduplicative structure was more readily recognized. An inspection of the looking behavior, however, suggested that, as soon as participants re-read the left words, the doubling preference emerged, and this was the case already within the first 500 ms of reading.

Altogether, the results of Experiment 2 show that, when doubling signaled licit plurals, the response to doubling shifted from aversion (to bare phonological forms, in Experiment 1) to preference (in Experiment 2). Importantly, this shift only occurred if a morphological parse was viable (i.e., when doubling was associated with a set of objects of the same kind): illicit plurals (sets of heterogeneous objects) are not amenable to a plural interpretation, and indeed, no hint of a doubling preference was found.

This shift in online doubling preferences is consistent with the predictions laid out by the abstract linguistic hypothesis. In particular, the hypothesis that linguistic responses are constrained by the abstract representation explains why a single physical stimulus can lead to divergent doubling preferences (i.e. aversion and preference). Furthermore, if this abstract knowledge guides natural language processing, then we would expect it to be deployed on-line, in early looking behavior. This is precisely what we found.

But do these findings falsify the alternative embodiment explanation? This question is harder to answer unequivocally. As noted in the Introduction, the embodiment account can acquire multiple forms. A strong version denies that abstract linguistic preferences exist; a weaker embodiment hypothesis admits that abstract constraints exist, but denies that they guide online language processing. Our results challenge both hypotheses.

A rejoinder, however, might point out that, although a single embodiment pressure cannot capture the entire pattern of results, these findings could potentially arise from two distinct sets of pressures. The doubling aversion (in Experiment 1) could potentially arise from a generalized form of repetition blindness (e.g. Kanwisher, 1987). The doubling preference (in Experiment 2), in turn, could arise from iconicity. Perhaps participants reason that “if one object is an X, then many objects of the same kind correspond to XX”.

We see several problems with this “mixed strategy” explanation. First, repetition blindness typically emerges only in serial visual presentations, and only under strict temporal parameters (typically, less than 250 ms for visual and auditory stimuli; Kanwisher, 1987); in our experiments, however, doubling was presented simultaneously, in a single word. Second, if repetition blindness were to play a role in Experiment 1, then why did it spare the right words in Experiment 2? Third, why did this metalinguistic iconic strategy constrain looking behavior already within the first fixation?

Other challenges to the iconicity strategy are presented by past research.

If doubling preferences arise from iconicity, then these preferences should arise universally, irrespective of speakers’ linguistic experience. Past research, however, shows this is not the case. Licit plurals elicited no doubling preferences in speakers of Mandarin—this is in line with the fact that their morphology does not productively mark plurality (Berent et al., 2020a, 2020b). Furthermore, Hebrew speakers showed a reliable doubling preference for diminutives (X = one ball; XX = small ball); this, too, is contrary to iconicity, but in line with the morphology of their language, where doubling marks diminution (Berent et al., 2016; Berent, Bat-El, & Vaknin-Nusbaum, 2016).

The challenge for the embodiment hypothesis, then, is to explain (a) why speech and signs elicit the same doubling preferences; (b) why doubling preferences depend on linguistic experience; and (c) why they arise online. At present, we cannot rule out the possibility that the embodiment view can meet these challenges. These questions, however, can all be readily addressed by the hypothesis that linguistic preferences are abstract.

## Description of Included files

- *Experiment 1–Phonology–Item Analyses:* Analyses by items for Experiment 1, the phonological condition. Includes raw data, item means, ANOVAs, and effect sizes.
- *Experiment 1–Phonology–Subject Analyses:* Analyses by participants for Experiment 1, the phonological condition. Includes raw data, participant means, ANOVAs, and effect sizes.
- *Experiment 2–Licit–Item Analyses:* Analyses by items for Experiment 2, the licit (morphological) condition. Includes raw data, item means, ANOVAs, and effect sizes.
- *Experiment 2–Licit–Subject Analyses:* Analyses by participants for Experiment 2, the licit (morphological) condition. Includes raw data, participant means, ANOVAs, and effect sizes.
- *Experiment 2–Illicit–Item Analyses:* Analyses by items for Experiment 2, the illicit (matched non-morphological) condition. Includes raw data, item means, ANOVAs, and effect sizes.
- *Experiment 2–Illicit–Subject Analyses:* Analyses by participants for Experiment 2, the illicit (matched non-morphological). Includes raw data, participant means, ANOVAs, and effect sizes.

## Data Availability

All data and analyses referenced in the paper can be accessed via the Open Science Framework at: https://osf.io/gtbk7/?view_only=15ec79b5124c4e029a0c38b3dfe3d854, https://doi.org/10.17605/OSF.IO/GTBK7.

## Appendix 1: Word stimuli for Experiments 1 and 2

**Table:**

Doubling (XX)	Control (XY)
glavlav	glavdap
slavlav	slavnag
glanlan	glanvap
plaflaf	plafsav
slanlan	slanvak
drakrak	drakmav
klaflaf	klafpar
smalmal	smalgar
kravrav	kravlan
snarnar	snarkal
prafraf	praftak
drafraf	drafpag
gravrav	gravlat
smavmav	smavgar
smafmaf	smafkal
brafraf	brafgat
snafnaf	snafgab
blavlav	blavmar
dravrav	dravkam
bravrav	bravgat
frasras	frasmal
slaflaf	slafmak
fralral	fralgad
blaflaf	blaftak
flaslas	flaspar
smarmar	smarvak
trafraf	trafkam
travrav	travgam
glatlat	glatrab
snavnav	snavmak

## Appendix 2: Detailed data processing method

### Data Pre-processing

The initial data generated by the eye tracker consisted of ordered sets of eye movement samples, with one set for each trial, resulting in 60 sets in Experiment 1 and 30 sets for each condition in Experiment 2. Each set was made up of three time-locked lists of equal length: the first was a list of time points, and the latter two were lists of (x, y) coordinate pairs, with one pair collected for each eye. Taken together, these lists represented the gaze location in screen coordinates for each eye, taken at each sampled time point. Because the Tobii EyeX has a sampling rate of 60 Hz, this meant that for each eye there were 60 records collected for every second’s worth of data. Because trials were self-paced, the total length of the eye movement records for each trial varied, but due to the five-second time limit, no trial contained more than 5 s worth of data.

The stimulus screen was divided into several regions of interest. These regions of interest served as the basic units of analysis for all subsequent data processing. They included the two word stimuli (left and right); the area of the screen containing the novel object; and the area occupied by the text prompt (“What is the best name for this object?”). For the purposes of the analyses described in this paper, the most important areas of interest were the two word-regions, *left word* and *right word*.

### Data Processing

Next, the gaze X and Y coordinates were categorized according to the defined regions of interest, so that for any given time point, a gaze location was assigned to one of the mutually exclusive regions of interest. The location of the participant’s gaze was approximated by averaging the coordinates for the two eyes. For time points that had data for only one eye (i.e. the other eye’s data was lost), the coordinates of the intact eye were used. Time points that lacked data for both eyes were classified as lost fixations (this usually occurred as a result of eye blinks but could also result from sub-optimal calibration). Then, the gaze’s location was classified according to which region of interest it fell into.

Once every data point for each trial and participant had been classified exhaustively according to the different regions of interest, the classified data was used to extract several measures of looking behavior. Three of these measures (the ones that are the focus of this paper) are described in more detail below. Because we were primarily interested in early processing, all data analyzed in the present paper strictly concern looking behaviors that occurred within the first three seconds of a given trial—any data collected after three seconds (trials could last as long as five seconds) were ignored for the purpose of these analyses. Most data fell into this category (> 95% of data across subjects occurred with the first three seconds of a trial).

## Appendix 3: Definitions of Measures Used in the Experiments

### Eye Movement Measures

*First fixation durations* The first measure collected was the average duration of the first fixation to each word in milliseconds. Fixations were defined as any look to a given word that lasted longer than 100 ms, and were determined from the data using an area-of-interest based algorithm (see Salvucci & Goldberg, 2000 for a discussion of how this method compares with other common fixation-identifying procedures). The cutoff of 100 ms was selected in order to capture the likely low end of the fixation duration distribution, based on typical fixation characteristics during reading as discussed elsewhere in the reading and eye movement literature (for a review of the typical range and average length of fixations during various reading-related activities, see Rayner, 1998). In this definition of fixations and in all subsequent measures of looking time, we ignore brief interruptions (lasting 80 ms or less, or the equivalent of 5 eye tracker samples) and count the duration of the interruption as part of the looking event itself.

*Total looking time.* Total looking time was defined as the average amount of time spent looking at a given word region over the entire course of a trial. Total looking times were calculated by adding up the durations of all looking events made toward a word region for each trial and dividing them by the number of trials. These looking events included first fixations, regressive fixations and looks that were shorter than a fixation (less than 100 ms).

*Regressive fixations.* Lastly, we examined the average number of regressive fixations, which we defined as the number of fixations that participants made to a given word region *after* the first fixation to that word. For example, if a participant fixated a given word three times during a given trial, the last two fixations were counted as regressive fixations. Importantly, regressive fixations only included looks that met the criterion for fixation (i.e. looks that were longer than 100 ms).

## Footnotes

1 The reading literature shows that fixations typically last 200 ms (Rayner, 1998)). Informed by this observation, we set the lower bound of “fixations” to 100 ms, as it is unlikely that shorter looks constitute fixations.
